# Pathogenesis and Mechanism of Uremic Vascular Calcification

**DOI:** 10.7759/cureus.64771

**Published:** 2024-07-17

**Authors:** Yingjing Shen

**Affiliations:** 1 Nephrology, Shanghai Tianyou Hospital, School of Medicine, Tongji University, Shanghai, CHN

**Keywords:** cell communication, uremic toxic substance, mechanism, chronic kidney disease-mineral and bone disorder, chronic kidney disease, vascular calcification

## Abstract

This review elucidates the modeling and mechanistic studies of vascular calcification in chronic kidney disease - mineral and bone disorder. In patients with chronic kidney disease, metabolic abnormalities in uremic toxins, including phosphate and indole sulfate, are closely associated with vascular calcification. Vitamin K, vascular circadian clock, and autophagy are also key factors involved in vascular calcification. Furthermore, communication between endothelial cells and smooth muscle cells also plays a pivotal role in the regulation of this process. Together, these factors accelerate vascular calcification progression and increase the risk of cardiovascular events. Therefore, timely intervention for vascular calcification is essential for patients with chronic kidney disease.

## Introduction and background

Chronic kidney disease (CKD) is a global public health concern with increasing prevalence and adverse consequences, including progressive loss of kidney function, cardiovascular disease, and premature death. For the first time in 2006, the Kidney Disease: Improving Global Outcomes (KDIGO) introduced the concept of chronic kidney disease - mineral and bone disorder (CKD-MBD). According to KDIGO, CKD-MBD is defined as a systemic mineral and bone metabolism disorder caused by CKD characterized by one or more of the following: abnormal metabolism of calcium, phosphorus, parathyroid hormone (PTH), or vitamin D; abnormal bone turnover, mineralization, volume, linear growth, or strength; and calcification of vasculature or other soft tissues. Vascular calcification (VC) in patients with CKD refers to ectopic deposition of calcium phosphate salts in the vascular wall and heart valves, including the coronary artery, abdominal aorta, iliac artery, and femoral artery. Epidemiological studies have demonstrated that ischemic heart disease, sudden death, arrhythmias, heart failure, stroke, and peripheral arterial disease account for more than 50% of the causes of late mortality in CKD [1]. CKD patients with cardiovascular calcification (CVC) are at increased risk of cardiovascular mortality [2]. The annual incidence of aortic valve calcification is close to 3.3% in patients on dialysis, and the prevalence of aortic and mitral valve calcification varies between 25% and 59%. Furthermore, the prevalence of VC is eight-fold higher in patients on hemodialysis (HD) than in the general population [3].

## Review

Clinical characteristics of VC in CKD-MBD

Vascular intimal calcification is often localized and is associated with atherosclerosis caused by lipid deposition and endothelial cell (EC) damage through inflammatory cell infiltration [4,5]. This calcification process resembles endochondral ossification as it involves both osteoblasts and chondrocytes, including progressive mineralization of cartilage matrix precursors or primordia [6]. The development and progression of VC are primarily influenced by genetic and lifestyle risk factors such as hypertension, diabetes, hypercholesterolemia, and smoking [7]. Atherosclerotic intimal calcification is linked with luminal stenosis and may lead to plaque rupture. It is a marker of atherosclerotic plaque burden and a strong predictor of cardiovascular events and mortality [8,9]. Vascular medial calcification can occur independently or concurrently with atherosclerosis and was originally referred to as “Monckeberg sclerosis.” This calcification is similar to intramembranous osteogenesis and driven by the mineralizing activity of osteoblasts without chondroid involvement [6]. Vascular medial calcification can lead to arterial stiffness, elevated pulse pressure, and increased pulse wave velocity, leading to left ventricular hypertrophy, dysfunction, and heart failure [4]. It mainly affects arteries that are less likely to develop atherosclerosis, such as the visceral abdominal aorta, thyroid arteries, pulmonary arteries, extremity arteries, and femoral arteries, which are more typical in CKD-MBD [10]. Calciphylaxis, also known as calcific uremic arteriolopathy, is an uncommon and very severe small arterial intima-media calcification commonly found in patients with renal failure afflicted with calcium and phosphorus metabolism disorders [11]. Lee et al. reported that vascular smooth muscle cells (VSMCs) from the peripheral arteries of patients with severe CKD undergoing amputation exhibited prominent characteristics of calcification and had significantly increased pathological calcification over time compared with those from normal human aorta [12].

Evolution of CKD-MBD animal models

The pathogenesis of CKD-MBD is complex and involves various feedback regulations among phosphate, calcium, PTH, vitamin D, and other factors [13]. Hence, an appropriate animal model is urgently warranted to elucidate the pathogenesis, pathophysiology, and molecular mechanism of CKD-MBD to provide novel insights into the development of new clinical treatment strategies [7]. There are currently three common methods for inducing CKD in animals, namely 5/6 nephrectomy, nephrotoxic drug-induced (calcitriol, adenine, and phosphate), and genetic engineering (autosomal-dominant polycystic kidney). The traditional animal model of atherosclerosis is established by feeding animals with a high-cholesterol and high-fat diet. To model hyperlipidemia and atherosclerosis, rabbits are the most commonly utilized animals. Owing to the unique features of vasculopathy in CKD-MBD, a high-fat diet alone is insufficient to recapitulate the pathophysiological process of CKD-MBD in CKD animal models. The selection of the appropriate animal species is a vital step in the construction of an animal model. Animal species vary in physiological and metabolic characteristics, presenting different advantages and limitations for the construction and study of specific disease models. The animal species used to study CKD-MBD are predominantly rats, followed by dogs, cats, and rabbits. However, rabbit kidneys are resistant to adenine toxicity and cats rarely develop CKD-related osteodystrophy [14].

Researchers have initially underscored on assessing whether the animal models exhibit elevated levels of phosphorus and PTH levels. Numerous studies have revealed that a high-phosphorus diet can successfully induce elevated PTH levels and parathyroid hyperplasia [15,16]. Later, researchers found that patients with CKD often present with extensive bone histologic changes, including mineralization and abnormal bone turnover, resulting in decreased bone mass and strength. Gagnon et al. first established a mouse model of stable CKD via nephrectomy and electrocauterization of the contralateral kidney in 1987. This model demonstrated biological and skeletal alterations that were similar to those observed in patients with CKD-MBD [17]. However, the model was not stable as the findings from bone pathology were inconsistent with those from bone micro-CT three-dimensional reconstruction. Moreover, mice are small animals with a limited blood volume and a high mortality rate [14]. An adenine diet (0.2% adenine + 1.8% phosphorus) has been demonstrated to induce rapid and remarkable changes in the alveolar bone of CKD mice, including enamel loss, increased dentin, decreased pulp, and severe alveolar bone resorption [18]. A study by Zhang et al. compared the effect of an eight-week low-calcium and high-phosphorus diet (0.6% calcium and 1.2% phosphorus) in the 5/6 nephrectomy model, adriamycin model, and ureter ligation model. Reduced calcium and elevated phosphorus, PTH, osteocalcin, and C-terminal telopeptide of type I collagen levels are observed in all three models. Three-dimensional micro-CT reconstruction of the femur revealed that the adriamycin model had the most severe distal femur bone destruction [19].

There has been a growing interest in VC in CKD-MBD in recent years. Since a high calcium level promotes calcium salt deposition in the vascular wall, many animal models have incorporated a high-calcium diet in addition to a high-phosphorus diet. Some models even include calcitriol to enhance calcium absorption, aiming to induce severe VC [20,21]. However, among the various promoters of VC in CKD-MBD, hyperphosphatemia (HP), but not hypercalcemia, is considered a key unconventional risk factor associated with uremia for VC in patients with CKD. A high-phosphorus diet of an appropriate prolonged duration is sufficient to induce thoracic aortic calcification, mitral valve calcification, and cardiac dysfunction in rats after 5/6 nephrectomy [22,23]. The adenine diet-induced model also plays an important role in the study of VC in CKD-MBD. The main advantage of this model is the avoidance of surgery and its associated complications. The adenine model was first established in rats, where the formation of adenine metabolite crystals in the proximal tubules leads to CKD development. Subsequently, this model was constructed using mice with a modified diet containing casein to maintain appetite [24]. Tölle et al. fed female DBA2/N mice with an adenine diet containing 2% adenine, 6% protein, 1% calcium, and 1% phosphorus and observed gradual calcification of the vascular walls of the abdominal aorta, renal artery, and most prominently the thoracic aorta starting from week 9 [25]. Similarly, Zhang et al. noted large-vessel calcification in the adenine mouse model, along with calcification of the heart valve and glomerular and tubular basement membranes. More importantly, X-ray energy spectrum analysis demonstrated that the composition of calcium-phosphorus nanocrystals in calcified blood vessels in mice was consistent with that in calcified human vascular tissues, indicating that this model is valuable for exploring calcification associated with CKD [22,26].

The 5/6 nephrectomy and adenine-induced nephropathy mouse models have been utilized to study CKD-related sarcopenia. However, researchers suggest that the body weight and composition changes in both models are primarily caused by energy deficit rather than renal dysfunction. Hence, mice were able to fully regain their initial lean body mass without displaying signs of emaciation or muscle atrophy. Thus, these two models may not be suitable for studying the mechanisms and treatment strategies of CKD-related sarcopenia [24].

The evolution of CKD-MBD animal models shows the ongoing advancement and refinement of scientific research. Through continuous optimization of animal models, researchers can more accurately simulate and study CKD-MBD, thereby providing better theoretical and experimental support for the prevention, diagnosis, and treatment of the disease.

Mechanism of VC in CKD-MBD

Phosphate

Among all drivers of VC, HP is the most important unconventional risk factor associated with uremia for VC in patients with CKD [7]. In patients with CKD, CVC is an early manifestation and its progression is linked with HP. HP is involved in several mechanisms that promote and trigger CVC [27]: 1. transformation of contractile VSMCs into an osteogenic/chondroid phenotype and mineralization of extracellular matrix; 2. induction of VSMC apoptosis; 3. transformation of monocytes/macrophages into bone resorption-like cells; 4. elevation of fibroblast growth factor 23 (FGF23); 5. downregulation of Klotho expression. One of the most critical events is the transformation of VSMCs into osteoblast/chondroblast-like cells. This transdifferentiation process is known as “re-programming” and is characterized by the loss of smooth muscle cell (SMC) markers such as α-smooth muscle actin (α-SMA) and the de novo expression of osteochondral markers such as Runt Related Transcription Factor 2 (RUNX2), alkaline phosphatase (ALP), osteopontin (OPN), and osteocalcin [28,29]. 

Saturated or near-saturated inorganic phosphorus (Pi) and calcium concentrations, along with a matrix for crystal nucleation, are the two essential conditions for initiating calcium phosphate precipitation and crystallization [30]. Calcium phosphate crystals in VSMC-derived extracellular vesicles (EVs) cause mineralization of the extracellular matrix during VC [28]. Phosphate, calcium, and calcium phosphate nanoparticles can activate pro-calcification signaling in cells. Pi levels are regulated by calcification inhibitors such as fetuin-A, which is produced by the liver. Fetuin-A binds with calcium and Pi to form a colloidal calcium phosphate complex known as the calciprotein particles (CPPs). CPPs can be further classified as primary and secondary CPPs, which differ in shape, function, and diameter [28]. Secondary CPPs induce vascular inflammation and osteogenic differentiation of myoblasts, ultimately abrogating the potential benefit of the colloidal calcium phosphorus complexes in the plasma [30]. An in vitro experiment has demonstrated that secondary CPPs can induce VSMC calcification and TNF-α expression and release, representing a promising novel biomarker and potential therapeutic target for VC [28]. The calcification propensity assay, based on the serum CPP maturation time (T50), was proposed to measure the time difference in transition from primary CPPs to secondary CPPs with hydroxyapatite. It is a novel in vitro blood test that provides a propensity score for additional extraosseous calcification [27]. Serum calcification propensity has been proposed as a biomarker for cardiovascular disease and has been demonstrated to predict cardiovascular disease and all-cause mortality in patients with CKD [29]. 

Inorganic pyrophosphate (PPi) is the strongest endogenous inhibitor of mineralization. Intracellular adenosine triphosphate (ATP) is transported to the extracellular space by ABCC6, a transporter. Hydrolysis of ATP by exonucleotide pyrophosphatase/phosphodiesterase 1 (ENPP1) leads to the generation of PPi, which inhibits the formation of hydroxyapatite. Conversely, PPi metabolism by exonucleotide triphosphate diphosphate hydrolase 1 (ENTPD1) and tissue nonspecific ALP increases the formation of Pi and HAP. As a result, the ENPP1/ENTPD1 ratio is a key regulator of Pi and PPi synthesis from ATP in SMCs [30].

Pi acts as a secondary intracellular messenger that activates several molecular pathways associated with bone formation [7]. Both in vitro and in vivo experiments showed that a high Pi level downregulates miR-145, which may be an early biomarker for VC and provide information on the initiation of VSMC transdifferentiation [31]. HP may directly increase TNF-α synthesis and secretion in monocytes via the Pit-1 pathway, leading to heightened systemic inflammatory responses and ultimately exacerbated VC [32]. Under hyperphosphatemic conditions, transcription factor EB (TFEB) becomes insoluble and is degraded via the ubiquitin-proteasome pathway. TFEB downregulation in turn leads to VC in VSMCs [33]. It is important to note that phosphatidylinositol is a key phospholipid signaling molecule for intravascular fistula formation. Phosphatidylinositol-3 kinase/AKT/mTOR signaling may be associated with a decreased inflammation, migration, and proliferation of SMCs. Platelet-derived growth factor (PDGF) is the most powerful mitogen and can trigger a “phenotypic transition” from a contractile state to a proliferative state, a key step in neointimal hyperplasia. PDGF-induced VSMC proliferation has been reported to be mediated by PI3K/AKT signaling [34]. Table 1 shows references in this section [35-52].

**Table 1:** Studies on HP-induced smooth muscle cell calcification models HP, hyperphosphatemia.

Drug	Signaling Pathway	Effect
Aldosterone	Inhibits AMPK-mediated autophagy	Promotes
Daprodustat	Activation of HIF-1 signaling	Promotes
NRF2 degradation	OGT-mediated KEAP1 glycosylation	Promotes
SGK3	Pit-1 signaling	Promotes
Empagliflozin	NFR2/HO-1 anti-inflammatory pathway through AMPK activation	Attenuates
Piperlongumine	Preserving P53/PTEN signaling	Attenuates
Zingerone	AMPK-mediated TIMP4 expression	Attenuates
RTEF-1	Wnt/β-catenin signaling pathway	Attenuates
β-glycerophosphate	Activating BK channels through Akt signaling	Attenuates
ASARM peptide	Inhibits expression of gut Npt2b	Attenuates
Dihydromyricetin	AKT signaling	Attenuates
GALNT3	Inhibits wnt/β-catenin signaling pathway	Attenuates
Globular adiponectin	PI3K/AKT and Wnt/β-catenin pathway	Attenuates
Intermedin1-47	WNT/β-catenin pathway	Attenuates
Kalkitoxin	RUNX-2 signaling pathway	Attenuates
Phosphonoformic acid	Pit-1 signaling pathway	Attenuates
Vitamin D	Prevents reduction in microRNA-145	Attenuates
O-GlcNAc transferase	Downregulating YAP	Attenuates

Other Uremic Toxins

Uremic toxins include small molecules (phosphate, trimethylamine N-oxide [TMAO]), large molecules (FGF23 and cytokines), and protein-bound toxins (indoxyl sulfate [IS] and methyl phenyl sulfate). Both small and large uremic toxins and especially protein-bound uremic toxins predispose CKD or HD patients to uremic VC [53]. Uremia promotes intestinal dysbiosis and disrupts the intestinal epithelial barrier [54], thus allowing live bacteria, endotoxins (lipopolysaccharide), and gut-derived uremic toxins to enter the systemic circulation, a condition commonly known as “leaky gut” [55]. In CKD, the pathogenesis of a leaky gut is a multifactorial process driven by sympathetic hyperactivity, intestinal congestion, hypogonadism, increased urine ammonia and ammonium hydroxide exposure, and decreased butyrate production [55]. The gastrointestinal tract is the source of uremic toxin production. Dietary aromatic amino acids are deaminated and decarboxylated by colonic microbes into p-cresol and other phenolic compounds, which are converted into p-cresyl sulfate (P-CS) by liver and colonic mucosa detoxification [56]. Dietary tryptophan is converted by the microbiota into indole, which is metabolized to IS by the liver [56]. TMAO is metabolized from substrates with high levels of trimethylamine, such as L-carnitine, choline, and betaine [54].

The most abundant type of uremic toxins is serum protein-bound uremic toxins, such as IS. Owing to its high affinity to plasma albumin, IS cannot be effectively removed by conventional HD. High P-CS and IS levels are associated with accelerated CKD progression and cardiovascular disease. IS concentration is 100-fold higher in patients with CKD than in healthy individuals. TMAO has been shown to promote vascular inflammation and VC through the activation of inflammasomes and induction of NF-κB expression. Advanced glycation end products (AGEs) also induce VC by upregulating AGE receptor expression, increasing oxidative stress, and triggering VSMC apoptosis [54]. In patients with CKD, plasma IS concentration is linked with cardiovascular events and mortality including markers of VC and atherosclerosis. It has been reported that IS promotes the pro-oxidative, pro-inflammatory, and pro-thrombotic processes of endothelial dysfunction, leading to atherosclerosis, impaired vascular repair, and thrombosis [57].

Prolonged vascular EC exposure to uremic toxins increases NADPH activity and oxygen free radical activity and downregulated levels of key antioxidant enzymes such as nitric oxide synthase and superoxide dismutase, resulting in reduced nitric oxide bioavailability and increased oxidation [53,58]. Another effect of uremic toxins on ECs is the induction of matrix metalloproteinases 2 and 9 and the downregulation of tissue metalloproteinase inhibitors 1 and 2, leading to increased extracellular matrix degradation [53]. IS is not only toxic for ECs but also for VSMCs. Both IS and phosphorus can induce IL-8 secretion by ECs, which downregulates OPN and promotes calcium salt deposition in VSMCs [59]. IS promotes Pit-1 expression and facilitates osteoblast-like differentiation and matrix mineralization of VSMCs through the activation of the JNK pathway [60]. It has been demonstrated that the PI3K/Akt/NK-κB signaling pathway plays an important role in IS-induced osteogenic transformation of VSMCs [61]. IS can also inhibit the activity of matrix Gla protein (MGP) via the ROS/NF-κB/miR-155-5p signaling pathway to induce osteogenic transdifferentiation of VSMCs [62].

Targeted removal of serum protein-bound uremic toxins is a crucial strategy for the management of uremic VC and can be attained through several approaches. First, timely removal of harmful substances and prevention of gastrointestinal toxin absorption facilitate gastrointestinal cleansing. Second, optimization of dietary composition and prebiotics or probiotics may help modulate the gut microbiota [63]. Third, the administration of oral toxin adsorbents may be effective against protein-bound uremic toxins. Fourth, a novel non-phosphate binding agent tenapanor has been demonstrated to reduce the paracellular and active transport of phosphate in the gut and increase fecal phosphorus levels while reducing urinary excretion of phosphate [64]. Finally, increased intestinal permeability is caused by altered zonulin expression. Hence, the use of the zonulin antagonist larazotide may improve the integrity of the intestinal barrier, thereby reducing the absorption of toxins [55]. Given that numerous vasotoxic compounds are derived from gut microbiota-mediated metabolism, the gut is increasingly recognized as a promising target for therapy [57].

Vitamin K

MGP is a vitamin K-dependent protein involved in VC inhibition. Interestingly, MGP must undergo two post-translational modifications to be active: γ-glutamic acid carboxylation and serine phosphorylation. Carboxylated and phosphorylated MGP is the active form of MGP that can inhibit VC by binding to calcium, hydroxyapatite, and bone morphogenetic protein-2 [65,66]. Vitamin K is a family of lipid-soluble molecules consisting of a 2-methyl-1,4-naphthoquinone ring. There are three major forms of vitamin K, which are distinguished by a lipophilic side chain at position 3: vitamin K1 (phylloquinone) with a chlorophyll side chain, vitamin K2 (menadione-7) with a varying number of isoprenoid units, and vitamin K3 (menadione) without a side chain [67]. The primary physiological role of vitamin K is to act as a cofactor for γ-glutamyl carboxylase, facilitating the addition of carboxyl groups to Glu residues in proteins during γ-carboxylation. Gamma-glutamyl carboxylase oxidizes vitamin K to vitamin K epoxide by removing a proton from the Glu residue and then adding a CO. In these proteins, the novel carboxylated residues are referred to as the Gla domain. This process converts inactive (non-carboxylated) proteins into active (carboxylated) proteins and allows them to bind calcium. A critical physiological step in bone mineralization and VC resistance is adequate calcium binding [68]. The decarboxylated matrix protein dp-ucMGP is an independent predictor for VC and a risk factor for atherosclerosis and cardiovascular death [66,69,70]. Vitamin K deficiency may indirectly manifest as high serum concentrations of dp-ucMGP and three additional forms of MGP, namely dp-MGP, p-ucMGP, and p-cMGP [71].

Most clinical studies have found that patients on HD are often deficient in vitamin K [65,69]. There are several possible explanations for this observation: 1. reduced dietary vitamin K intake; 2. decreased expression and activity of vitamin K cyclo-oxidoreductase, which can recycle vitamin K to increase its tissue utilization; 3. oral administration of phosphate binders that prevent vitamin K absorption in the gastrointestinal tract [67].

Studies in recent years have investigated the necessity of vitamin K supplementation in patients with CKD. Some interventional studies demonstrated no clear benefit of vitamin K supplementation in VC associated with CKD [72]. Yet, others have suggested that exogenous vitamin K supplementation can prevent or even reverse VC, protect the cardiovascular system, and may serve as a prophylactic target for VC [72]. In patients with CKD, the therapeutic significance of vitamin K remains unclear, and large-scale and well-designed randomized controlled trials evaluating the impact of vitamin K supplementation on CKD are warranted to draw more definitive conclusions.

Vascular Circadian Clock

In CKD, the amplitude of melatonin rhythm declines as kidney function decreases. Additionally, dysregulated melatonin rhythms are associated with sleep disturbances in patients with CKD. Melatonin is mainly released from the pineal gland and is involved in the sleep-wake time, blood pressure regulation, and synchronization of the circadian rhythm (CR). The circadian clock is an endogenous, self-sustaining pacemaker that operates on a 24-hour cycle, coordinating the rhythms of metabolism, hormone secretion, cell cycle, inflammation, and cardiovascular function [73]. It has been demonstrated that each cell type in the blood vessel, such as ECs [74], medial VSMCs [75], and cultured adventitial fibroblasts [76], has its own functional circadian clock. In CKD, CR disruption has been identified in activin A, FGF23, PTH, and phosphate levels [73]. CR disruption may be involved in the early development of uremic vasculopathy, leading to the development of cardiovascular disease and serious health problems [76]. The molecular circadian clock within the central circadian pacemaker and peripheral tissues associated with CKD-MBD is dysregulated in CKD [73,76]. It was found that the expression of clock control genes linked with vascular integrity, endothelial function, inflammation, and thrombosis is severely dysregulated in the calcified aorta in CKD. This is evidenced by the significantly upregulated expression of Rev-Erba, Clock, Cry2, and CK1# and the downregulated expression of Per1. Egstrand et al. revealed that uremia disrupts the intrinsic molecular clock in the rat aorta. Other genes governing the CR of platelet activation, fibrinolytic activity, and coagulation may serve as a link between the intrinsic vascular circadian clock and the temporal pattern of cardiovascular disease, and dysregulation of these genes could potentially lead to calciphylaxis [77]. Temporal therapy may be an important approach for treating CKD-MBD in the future, given the potential role of the circadian clock in CKD-MBD.

Autophagy

Autophagy is an important biological process that serves as a garbage-removal system within cells. This process enables cells to remove or degrade damaged or unwanted organelles and proteins, thus playing a pivotal role in maintaining intracellular homeostasis, responding to stress, and regulating metabolism. There is increasing evidence showing that autophagy helps maintain normal vascular cell function and exerts a protective effect against VC. Numerous studies have indicated that autophagy is involved in the dedifferentiation of VSMCs to mesenchymal stem cells and promotes the conversion of calcification signals during vascular wall mineralization. While autophagy is protective under physiological conditions, pathological autophagy may lead to excessive calcification [78]. In animal models of CKD, Brahma-related gene 1 (BRG1) [79], histone deacetylase 2 (HDAC2) [80], Irisin [81], and β-hydroxybutyrate [82] have all been demonstrated to inhibit VC and protect SMCs through activation of autophagy. Therefore, VSMC autophagy regulators may provide novel targets for the treatment of VC.

Communication Between ECs and SMC

EVs derived from ECs constitute a major type of EVs. They are involved in the regulation of numerous physiological functions, particularly intravascular homeostasis. EC-derived EVs can interact with various types of vascular cells and carry a broad range of biological molecules that stimulate various intracellular pathways linked with angiogenesis, neovascularization, tissue regeneration, cytoprotection, and wound healing [83]. The mechanisms by which ECs and VSMCs communicate with EVs are still under active research.

A comparison of EC-derived and VSMC-derived EVs after stimulation by uremic toxins demonstrated that both types of EVs contain a common protein flotillin-1. Moreover, EC-derived EVs are positive for tumor susceptibility gene-101, whereas VSMC-derived EVs are positive for syntenin-1 [84]. Lano et al. explored the effect of EVs on transforming growth factor-β (TGF-β) signaling and VSMC proliferation. The authors stimulated human umbilical vein endothelial cells (HUVECs) with IS and found that the secreted EVs contained TGF-β, which stimulated the proliferation of aortic VSMCs in a concentration-dependent manner [85]. This indicates that EC-derived EVs are involved in the pathogenesis of vascular stenosis by modulating TGF-β signaling in uremic VSMCs [86]. The effect of EC-derived EVs on Wnt signaling and VSMCs has also been examined by stimulating HUVECs with HP. Qin et al. demonstrated that STAT1-rich exosomes secreted by HP-treated HUVECs are delivered to VSMCs, thus promoting VSMC calcification through the activation of Wnt/β-catenin signaling. This result indicates that HP-HUVEC-Exo has calcium-promoting effects [87]. In addition to target proteins, miRNAs encapsulated in EC-EVs have been demonstrated to modulate blood vessels in CKD-MBD, with miR-143-3p and miR-145-5p inhibiting VC and miR-221-3p and miR-222-3p enhancing VC [88]. 

Presently, mechanistic studies of the effect of vascular EC-derived EVs on VSMCs remain limited. Some limitations of these studies include the following: 1. There are many in vitro experiments but very few in vivo validation studies. 2. Most of the current in vitro models utilize HUVECs and aortic ECs. HUVECs are typically cultured in EC medium, whereas aortic ECs are cultured in high-glucose Dulbecco's Modified Eagle Medium (DMEM). Co-culturing these cells remains challenging due to the difference in culture media requirements. 3. Aortic ECs and aortic SMCs are adjacent cell layers that form the vascular wall. Thus, aortic ECs are a more relevant cell type for studying the associated pathophysiological mechanisms.

Treatments for VC in CKD-MBD 

CKD-MBD is a complex multifactorial syndrome that currently lacks effective treatment. Current conventional treatment strategies are more focused on delaying VC progression through phosphate balance, correction of vitamin D and vitamin K deficiencies, avoidance of high and low bone turnover, maintenance of normal serum calcium levels, and attenuation of inflammatory responses [89]. Therapies targeting bone and mineral metabolism may either slow or accelerate VC progression in patients with CKD [3]. Vitamin D deficiency has been implicated in endothelial dysfunction, increased vascular stiffness, hypertension, left ventricular hypertrophy, and congestive heart failure [1]. Hence, maintaining “normal” vitamin D levels may be beneficial for sustaining normal bone turnover and potentially delaying CVC progression [90]. Current guidelines recommend that patients with CKD should prevent vitamin D deficiency and initiate vitamin D supplementation before severe secondary hyperparathyroidism occurs [91]. Antiresorptive agents, such as bisphosphonates and romosozumab, may help alleviate VC while treating osteoporosis. Although sodium thiosulfate has demonstrated some efficacy in the treatment of calciphylaxis, evidence supporting its effectiveness in attenuating CVC is lacking. Several clinical trials have evaluated the effect of targeting VC directly with SNF472 (a novel phytate derivative that inhibits hydroxyapatite formation) and magnesium, or indirectly with sodium thiosulfate and vitamin K [9]. In addition to traditional pharmacotherapy, the emergence of novel approaches, such as targeted drugs and biological agents for VC, has offered new treatment options for patients with CKD-MBD.

## Conclusions

Uremic VC is indeed a significant complication in patients with CKD and end-stage renal disease. The pathophysiological mechanisms involved in this process are multifactorial and complex, as mentioned above. HP is a major driver of VC. Elevated phosphate levels can lead to the deposition of calcium phosphate crystals in the vasculature. Inflammation can trigger the release of cytokines and growth factors that stimulate VSMC differentiation into osteoblast-like cells. Programmed cell death, or apoptosis, can contribute to the release of calcifying vesicles. VSMCs can undergo a phenotypic switch to a more osteogenic phenotype, characterized by the expression of bone-related proteins and mineralization capacity, contributing to VC. Further research into these pathways and their interactions will be essential for advancing our knowledge and improving clinical outcomes for the affected individuals.
